# Amyloid cardiomyopathy presenting with gastrointestinal symptoms of abdominal pain, back pain, and constipation: A case report

**DOI:** 10.1097/MD.0000000000045049

**Published:** 2025-10-03

**Authors:** Yike Zhang, Tingting Zhang, Manli Zhao, Peichun Li, Jiangbo Xie

**Affiliations:** a Clinical Integration of Traditional Chinese and Western Medicine, Shandong University of Traditional Chinese Medicine, Jinan, China; b Department of Rehabilitation, Fujian MedicalUniversity, Fujian, China; c Weifang Hospital of Traditional Chinese Medicine, Shandong Second Medical University, Weifang, China.

**Keywords:** abdominal pain, amyloid cardiomyopathy, back pain, case report, constipation

## Abstract

**Rationale:**

Amyloid cardiomyopathy (AC) is a rare and fatal condition, often with a delayed diagnosis due to its nonspecific initial presentations. This case underscores the diagnostic challenge and critical importance of early recognition when patients present with seemingly unrelated gastrointestinal symptoms.

**Patient concerns:**

A 48-year-old woman initially presented with abdominal pain, back pain, and constipation, which were managed as gastrointestinal disorders. Her condition progressed rapidly to include severe cardiac symptoms, including chest tightness and dyspnea.

**Diagnoses:**

Cardiac magnetic resonance imaging revealed findings indicative of AC, including left ventricular hypertrophy, diffuse delayed enhancement, and atrial enlargement. The diagnosis of immunoglobulin light chain amyloidosis was confirmed by endomyocardial biopsy, which was positive for κ light chains.

**Interventions:**

Supportive care for heart failure, including diuretics and beta-blockers, was initiated. However, specific therapies targeting the underlying immunoglobulin light chain amyloidosis could not be administered due to the patient’s rapid clinical deterioration.

**Outcomes:**

The patient died 2 days after the pathological diagnosis was established, highlighting the aggressively progressive nature of the disease.

**Lessons:**

This case highlights that AC can manifest with prominent gastrointestinal symptoms before overt cardiac involvement. It is crucial to consider systemic amyloidosis in the differential diagnosis for patients with multisystem involvement, as early, multidisciplinary evaluation is essential for potentially improving outcomes in this fatal condition.

## 1. Introduction

Cardiac amyloidosis (CA) is a rare and often underdiagnosed condition characterized by the deposition of amyloid fibrils in the myocardial extracellular space, leading to restrictive cardiomyopathy and progressive heart failure.^[1]^ The 2 primary types of CA are light chain (AL) amyloidosis and transthyretin (ATTR) amyloidosis, with AL amyloidosis associated with a poor prognosis due to their rapid progression and significant hemodynamic impact.^[2,3]^

The clinical manifestations of CA are heterogeneous, ranging from asymptomatic to severe heart failure, and often includes symptoms such as dyspnea, peripheral edema, and arrhythmias.^[4]^ Diagnosis typically involves a combination of clinical suspicion, imaging techniques (e.g., echocardiography and cardiac magnetic resonance imaging [MRI]), and confirmatory tests (e.g., biopsy and scintigraphy).^[5]^

Here, we report the case of a 48-year-old woman with amyloid cardiomyopathy whose initial presentation was dominated by gastrointestinal symptoms (abdominal pain, back pain, and constipation), later progressing to severe cardiac manifestations. The rarity of this presentation highlights the diagnostic challenges of CA and the importance of early recognition.

## 2. Case report

**Figure 1.**
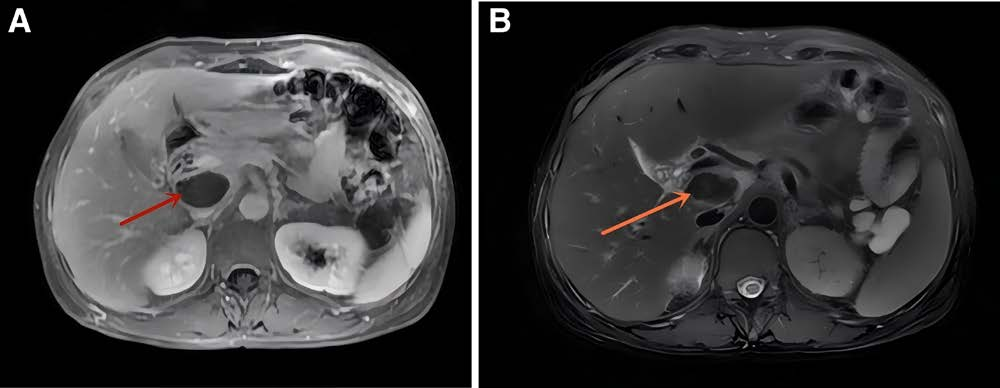
(A and B) MRI plain scan and enhancement were performed in the hepatic and biliary area showed abnormal signals in the portal vein and inferior vena cava space were considered as hematoma. MRI = magnetic resonance imaging.

**Figure 2.**
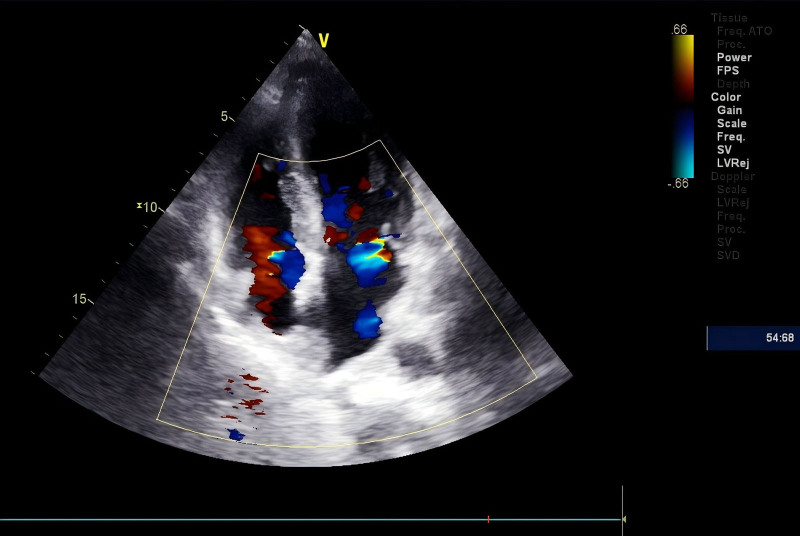
Color Doppler ultrasonography showed that the left ventricular wall was thickened, diastolic function decreased, both left and right atria enlarged, and atrial septum thickened.

A 48-year-old woman initially experienced pain in the upper right abdomen, nausea, acid reflux, and hiccups while doing housework 1 month prior to presentation. These symptoms relieved on their own after approximately 1 hour. Ten days before presentation, the symptoms recurred, accompanied by constipation. She reported bowel movements only once a week, despite no prior history of constipation. The patient visited a local clinic and was prescribed “mosapride tartrate” by the outpatient department and was advised to visit a general hospital for a clear diagnosis and treatment. After taking the medicine, the patient’s condition improved slightly, and did not continue to seek medical attention. One night before admission, she experienced sudden abdominal pain while sleeping. The pain intensified and persisted without subsiding, and was accompanied by back pain and discomfort. Emergency physical examination revealed the following: blood pressure, 110/70 mm Hg; heart rate, 88 beats per minute; mild tenderness in the upper abdomen; and no protective or rebound tenderness. The patient experienced significant abdominal pain at night. Emergency computed tomography scans of the upper and lower abdomen revealed a block-like lesion of slightly increased density within the common bile duct. The patient had a mild low-density lesion in S8 segment of the liver and had previously undergone cholecystectomy. Based on these findings, sediment-like bile duct stones, bile duct tumor, or hepatic space-occupying lesions were considered. She was admitted to the gastroenterology department of our hospital for further diagnosis and treatment. Plain and enhanced MRI scans of the hepatobiliary tract showed abnormal signals in the portal vein and inferior vena cava regions, suggesting a hematoma (Figs. 1A, B, and 2). Laboratory tests revealed normal liver enzyme (alanine aminotransferase, 45 U/L; aspartate aminotransferase, 38 U/L) and lipase (60 U/L) levels. Emergency electrocardiography showed sinus rhythm with T-wave inversion in leads II, III, avF, and V2 to V6. The patient initially had no symptoms of cardiac discomfort, and no further cardiac ultrasound examination was conducted. The retrospective medical history showed that the patient had a history of riding an electric vehicle 3 months prior, which was considered to be the cause of the hepatobiliary hematoma. Owing to the suspicion of biliary tract inflammation, ceftriaxone was empirically administered. During hospitalization, gastroscopy and colonoscopy revealed chronic nonatrophic gastritis with mucosal bleeding and erosion, as well as small colonic polyps. She received symptomatic and anti-infection treatment during hospitalization and was discharged with mild abdominal distension and discomfort.

**Figure 3.**
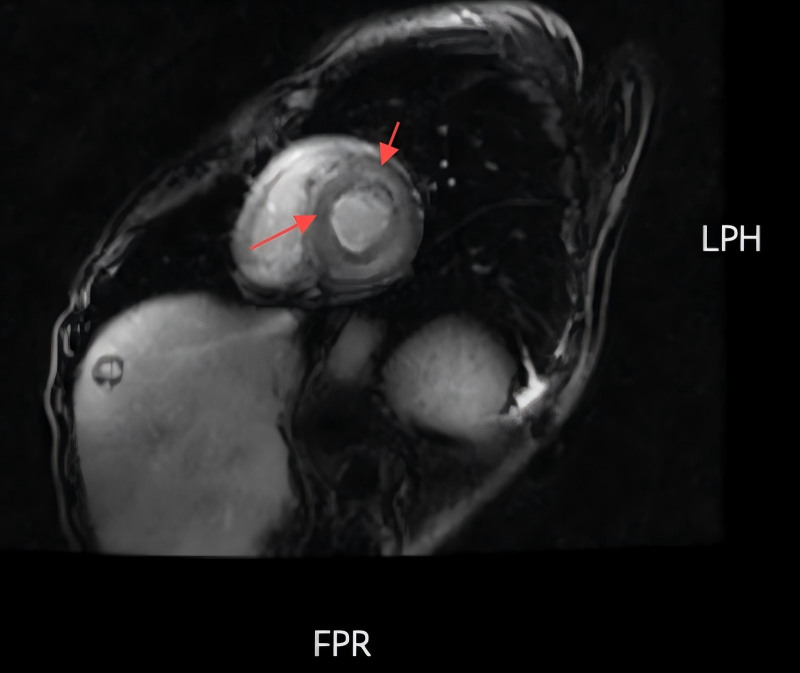
The cardiac magnetic resonance results showed double atrial enlargement, atrial septal thickening, ventricular septal and left ventricular wall thickening with diffuse delayed enhancement, left ventricular diastolic dyskinesia, and high suspicion of myocardial amyloidosis.

Two weeks later, she was admitted to the gastroenterology department with abdominal distension and constipation. No obvious digestive system abnormalities were observed during gastroenterological examination. Two days after hospitalization, she experienced chest tightness, nocturnal discomfort, and back pain. A physical examination revealed a blood pressure of 90/60 mm Hg, moist rales at the base of both lungs, and pitting edema of 2 + in both lower extremities. The electrocardiogram showed sinus rhythm with T-wave inversion in leads II, III, avF, and V2 to V6. Laboratory testing revealed markedly elevated N-terminal pro–B-type natriuretic peptide at 3472 pg/mL (reference range: <300 pg/mL) and hypersensitive troponin I at 86.7 pg/mL (reference range: 0–17.5 pg/mL). Cardiology consultation considered the possibility of coronary heart disease, and she was transferred to that department. No obvious abnormalities were found on the emergency coronary angiography. Color Doppler ultrasonography showed left ventricular wall thickening, decreased diastolic function, enlarged left and right atria, and a thickened atrial septum (Fig. 2, Table 1). Therefore, the possibility of cardiomyopathy was considered. The patient’s chest tightness and breathing difficulties worsened rapidly, making diagnosis difficult. The patient was then transferred to a high-level hospital (Table 2). Cardiac MRI at a superior hospital revealed bilateral atrial enlargement, thickened atrial septum, thickened ventricular septum, diffuse delayed enhancement of the left ventricular myocardium, and impaired diastolic function, findings highly suggestive of myocardial amyloidosis (Fig. 3). The pathological results revealed mild amyloidosis. Serum free light chain test showed that the κ/λ ratio increased by 12.5 (normal: 0.26–1.65). A myocardial tissue biopsy and histological examination revealed amyloid protein deposition. Congo red staining confirmed this, and apple-green birefringence was observed under polarized light. Immunohistochemistry showed positive κ light chain, supporting the diagnosis of AL amyloidosis. Electron microscopy confirmed the presence of amyloid fibrils. Unfortunately, because of the rapid clinical deterioration of the patient, mass spectrometric analysis was not performed. The patient died 2 days after the pathological examination.

**Table 1:** Summary: diagnostic findings in AL amyloid cardiomyopathy.

Category	Key findings
Imaging	- Cardiac MRI: left ventricular hypertrophy, diffuse delayed gadolinium enhancement, atrial enlargement - Echocardiography: diastolic dysfunction, thickened atrial septum
Biomarkers	- Pro-BNP: 3472 pg/mL (↑↑) - hs-Troponin I: 86.7 pg/mL (↑)
Histopathology	- Congo red staining: Apple-green birefringence (polarized light) - Immunohistochemistry: κ light chain positivity - Electron microscopy: Confirmed amyloid fibrils
Serology	- Serum free light chains: κ/λ ratio 12.5 (normal 0.26–1.65)
Limitations	Mass spectrometry not performed due to rapid deterioration

AL = immunoglobulin light chain, MRI = magnetic resonance imaging.

**Table 2:** Summary: clinical timeline and outcomes.

Timepoint	Clinical events
Initial admission	- Presenting symptoms: Abdominal/back pain, constipation - Misdiagnosed as biliary disorder - CT/MRI: Hepatic hematoma, gallbladder wall thickening
Readmission	- Progressive symptoms: Chest tightness, dyspnea, hypotension (90/60 mm Hg) - Elevated cardiac biomarkers - Coronary angiography: No obstruction
Final diagnosis	- Cardiac MRI + biopsy: Confirmed AL amyloidosis (κ+) - Death within 48 h of diagnosis
Therapeutic interventions	- Supportive care: Diuretics, β-blockers - No AL-specific therapy due to rapid decline

AL = immunoglobulin light chain, MRI = magnetic resonance imaging.

## 3. Discussion and conclusion

This case carries important clinical implications. Its core enlightenment lies in accurately identifying systemic AL amyloidosis in a patient who initially presented with gastrointestinal symptoms and in distinguishing it from isolated CA. The patient, a middle-aged woman, initially presented with intractable abdominal, back, and constipation pains. These symptoms persisted and conventional treatment was ineffective, suggesting the presence of systemic lesions rather than simple gastrointestinal diseases. Although κ light chain AL amyloidosis was eventually diagnosed through endocardial biopsy, retrospective analysis revealed that her clinical manifestations (involvement of multiple systems), laboratory tests (significantly elevated κ/λ ratio), and imaging features were more consistent with the characteristics of systemic AL amyloidosis.^[6,7]^

Systemic AL amyloidosis and isolated CA have significant differences in several aspects. First, systemic lesions often involve multiple organs (such as the kidneys, liver, gastrointestinal tract, and nervous system), while isolated lesions are limited to the heart. Second, the prognosis of the systemic AL type is even worse, with a 5-year survival rate of <30%^[8]^; most importantly, the treatment strategies for the 2 are completely different. Systemic lesions require systemic treatment led by the hematology department (such as chemotherapy or daratumumab),^[9]^ while isolated lesions mainly require cardiac function support. In this case, the diagnostic delay was largely due to the failure to promptly recognize these differences.

In terms of diagnostic strategies, we gained the following important experiences: In patients presenting with multiple systemic symptoms, the possibility of systemic amyloidosis must be given priority. The diagnostic process should be optimized. In accordance with the principle of “from noninvasive to invasive,” serum free light chain detection (sensitivity > 90%) and radionuclide imaging should be conducted first, followed by fat pad biopsy (positive rate 70%–80%) or gastrointestinal mucosal biopsy, and finally endocardial biopsy.^[7,10]^ A multidisciplinary approach involving gastroenterology, cardiology, and hematology is essential. It is particularly important to emphasize that speckle tracking echocardiography (STE) technology has unique value in noninvasive imaging. Latest research shows that STE can provide an important basis for the early diagnosis and prognostic stratification of amyloidosis by detecting the characteristic manifestations of longitudinal strain deterioration in the basal and middle segments while the apical segment is relatively preserved (the “apical preservation” pattern).^[11]^ Therefore, this technique should be promoted and applied in clinical practice to supplement routine echocardiography.

The lessons learned from this treatment are equally profound. However, the treatment window for systemic AL amyloidosis is limited. In this case, it only took 2 months from the onset of symptoms to death, indicating that: early consultation with the hematology department is of vital importance; early application of new therapeutic drugs such as daratumumab may improve prognosis^[9]^; and it is necessary to establish a rapid diagnosis and treatment channel.

The patient’s death within 2 days of histopathologic diagnosis not only reflects the perilous nature of AL amyloidosis but also exposes the deficiencies in the current diagnosis and treatment process. Following priorities should be addressed in the future: developing screening strategies for patients with multiple system involvement, establishing regional diagnosis and treatment centers for amyloidosis, and strengthening the training of non-hematologists to enhance their ability to identify atypical manifestations.^[7,9,12,13]^ In terms of technological applications, efforts should be made to actively promote standardized use of advanced imaging techniques such as STE and to incorporate them into the diagnostic and prognostic assessment system for amyloidosis.

In conclusion, the core value of this case lies in: clarifying the key points for differentiating systemic AL amyloidosis from isolated heart disease; proposing strategies to optimize the diagnostic process, with particular emphasis on the application value of advanced imaging techniques such as STE; and highlighting the critical importance of multidisciplinary collaboration. These findings have significant reference value for improving the diagnosis and treatment of amyloidosis.

## Author contributions

**Conceptualization:** Jiangbo Xie.

**Writing – original draft:** Peichun Li.

**Writing – review & editing:** Yike Zhang, Tingting Zhang, Manli Zhao, Jiangbo Xie.
